# PARP1 Exacerbates Prostatitis by Promoting M1 Macrophages Polarization through NF-κB Pathway

**DOI:** 10.1007/s10753-025-02247-y

**Published:** 2025-03-04

**Authors:** Lu Jin, Jiaxing Chen, Jianhui Fu, Jingyi Lou, Yingxue Guo, Xia Liu, Xiaojuan Xu, Huiying Fu, Qiyang Shou

**Affiliations:** 1 The Second Affiliated Hospital & Second Clinical Medical School, Jinhua Academy, Zhejiang Chinese Medical University, Hangzhou, 310053 China; 2 Department of Urology, Jiangshan People’s Hospital, Jiangshan, 324100 China; 3 College of Life Science, Zhejiang Chinese Medical University, Hangzhou, China; 4 School of Pharmacy, Zhejiang Chinese Medical University, Hangzhou, 310053 China; 5 Chun ‘an First People’s Hospital, Hangzhou, 310053 China

**Keywords:** PARP1, Prostatitis, M1 macrophages, Inflammatory cytokines, NF-κB

## Abstract

PARP1 is recognized for its role as a DNA damage sensor and its involvement in inflammatory diseases, but its impact on prostatitis remains unclear. We aimed to elucidate how PARP1 affects prostatitis progression. Our results showed that in 1% carrageenan-induced prostatitis mouse model, *Parp1*^−/−^ prostatitic mice showed less pathological damage, decreased prostate weight, and lower inflammatory indices, decreased macrophage and neutrophil infiltration, down-regulated the expression of pro-inflammatory cytokines (IL-6, IL-12p70, CCL2, TNF) and up-regulated anti-inflammatory cytokine IL-10 in prostate tissue. The expression of NF-κB, TNF, and IL-6 mRNA in the prostate tissue of *Parp1*^−/−^ prostatitic mice decreased. *In vitro* experiments revealed that M1(CD206^−^CD86^+)^ macrophage in LPS-induced macrophage of *Parp1*^−/−^ mice decreased, as did iNOS, TNF, IL-6 and NF-κB mRNA expression. Mechanically, treatment with the PARP1 inhibitor (AG14361) led to a significant reduction in NF-κB mRNA and Phospho-NF-κB P65 protein expression in macrophages. Following intervention with NF-κB inhibitors (Bay 11–7082), both IL-6 protein and mRNA levels were markedly diminished, meanwhile the secretion of IL-6, IL-10, IL-12p70, CCL2, IFN-γ, and TNF exhibited a pronounced dose-dependent decrease. Collectively, these findings indicated that PARP1 exacerbates carrageenan-induced prostatitis by promoting M1 macrophages polarization *via* the NF-κB pathway, suggesting PARP1 could be a potential therapeutic target for macrophage-based treatments in prostatitis.

**Supplementary Information:**

The online version contains supplementary material available at 10.1007/s10753-025-02247-y.

## Introduction

Chronic prostatitis/chronic pelvic pain syndrome (CP/CPPS), classified as either category IIIA or IIIB in the National Institutes of Health classification of prostatitis without bacterial infection, has been estimated to occur in up to 90% of patients with prostatitis [1, 2]. Owing to the absence of immediate severe clinical manifestations, this condition is frequently underestimated by patients. Nonetheless, the chronic inflammation associated with it can be particularly distressing for males with reproductive disorders, ultimately exerting a substantial negative impact on patients’ quality of life [3, 4]. The pathogenesis of chronic prostatitis is intricate and not yet fully understood. The absence of a reliable etiological diagnostic method results in suboptimal treatment outcomes and recurrent symptoms. Consequently, exploring the underlying mechanisms of prostatitis is essential for advancing future management strategies.

The infiltration of mixed inflammatory cells directly reflects the body’s continuous inflammatory response to injury and is a typical pathological manifestation of chronic prostatitis in clinical settings [5]. When the prostate tissue is infltrated by inflammatory cells, various active substances and chemokines are released [1]. Chronic prostatitis is characterized by the activation of the mononuclear macrophage system [6, 7], while neutrophils are activated in prostatitis leading to damage to normal tissues and exacerbating local inflammatory responses [8, 9]. Additionally, the role of cytokines as the main regulatory mediator in the process of prostatitis pathogenesis has received extensive attention [10]. Studies have shown that the therapeutic response is related to the expression of cytokines (*e.g.*,TNF-a, IL-6, IFN-γ, IL-2, IL-1β), compared with white blood cells, cytokines can respond to patients condition changes sooner and more accurately [11, 12].

PARP1 serves as an extensively distributed intracellular nuclear protease [13]. Numerous studies have demonstrated that the inhibition or knockout of PARP-1 effectively suppresses the expression of inflammatory mediators and downregulates various chemokines induced by chemical stimuli, including IL-8, macrophage inflammatory protein-1 (MIP-1), and monocyte chemoattractant protein 1 (MCP-1) [14, 15]. Moreover, PARP1 has been extensively implicated in regulating prostate cancer progression and development [16, 17], with concomitant presence of prostatitis presenting as a high-risk factor for prostate cancer. However, the precise role of PARP1 in the prostatitis remains unclear. Therefore, this study utilized a carrageenan-induced prostatitis model to investigate the impact and potential mechanism of PARP1 on the onset and progression of prostatitis.

## Materials and Methods

### Animal Experiments

PARP1 gene knockout (*Parp1*^−/−^, Strain Name:129S-*Parp1*^tm1ZqwjJ^, Stock Number:002779) mice aged 8 weeks were obtained from the Jackson Laboratory. After arrival at our facility, mice were crossed with wild-type C57BL/6 J mice for eight generations prior to use of the mice in the current analyses. We housed them under pathogen-free condition with a maximum of three mice per cage. We strictly adhered to animal care principles and ethics and recevied approval from the Animal Experimentation Centre Committee of Zhejiang Chinese Medical University.

The *Parp1*^−/−^ mice were genotyped by isolating DNA from their tails using a DNA extraction kit. The PCR reaction conditions included initial denaturation at 94 °C for 3 minutes, followed by 35 cycles of amplification at 94 °C for 30 seconds, annealing at 64 °C for 1 minute, extension at 72 °C for 1 minute, and final extension at 72 °C for 2 minutes. The primer sequences used were as follows: wild type reverse primer −5’-CCAGCGCAGCTCAgAGAAGCCA-3′, common primer −5’-CATGTTCGATGGGAAAGTCCC-3′, mutant reverse primer −5’-AGGTGAGATGAC

AGGAGATC-3′. The amplified product resulted in a fragment size of 350 bp.We strictly adhered to animal care principles and ethics and recevied approval from Ethics Committee of Animal Experiments at Zhejiang Chinese Medical University (approval number IACUC-20201019-02).

### Prostatitis Model

A chronic prostatitis mouse model was induced using 1% carrageenan (Sigma-Aldrich, Cat: C1138, USA) saline [18] All mice were anesthetized with isoflurane inhalation, and the lower abdominal hair was removed. Under aseptic conditions, a median incision was made in the lower abdomen straight to the abdominal cavity to expose the prostate gland. The model group was injected with 1% sterile carrageenan saline solution into the lateral lobes of both sides of the anterior of the prostate at the corresponding dose (5, 10, 20 μL) for each group, while the control group received saline injections. The mice were weighed daily and euthanized with CO_2_ after 7 days. Their prostate glands were then removed and weighed to calculate the prostate index and inflammatory prostate index. The prostate index (%) is calculated as total prostate weight (mg)/body weight (g) × 100%, inflammatory prostatic index (%) = inflammatory prostate weight (mg)/body weight (g) × 100%.

### Cell Experiment

Peritoneal murine macrophages were isolated from 8-week-old male *Parp1*^−/−^ and *Parp1*^+/+^ mice. The mice were intraperitoneally injected with 2 mL of 4% brewer thioglycolate medium, and the inflammatory response was allowed to proceed for 4 days. Macrophages were harvested from peritoneal lavage fluid, washed with PBS, and cultured in DMEM supplemented with 10% fetal bovine serum (FBS; Gibco). To establish macrophage inflammation models, cells were seeded into 24-well plates at a density of 5 × 10.^5^ Following overnight adhesion, the cells were exposed to LPS (100 ng/mL or 200 ng/mL) for 24 hours. The supernatant was collected for inflammatory factor (IL-6, IL-10, IFN-γ, TNF-a, and CCL2) level detection, and the cells were utilized for western blot and quantitative real-time PCR experiment. Simultaneously in the macrophage inflammation model, NF-κB protein levels were assessed following treatment with the PARP1 inhibitor (AG14361) at dose of 10 μM. Inflammatory factor expression were detected after NF-κB inhibitor (Bay 11–7082) treatment at dose of 12.5, 25 and 50 μM in LPS-induced macrophage model.

At the age of 8 weeks, male C57BL/6 mice were anesthetized with isoflurane for blood collection from the heart under sterile conditions. Neutrophils were isolated using the mouse peripheral blood neutrophil isolation KIT (Cat: LZS1100, Tianjin Haoyang Biological Manufacture Co., Ltd) according to the provided instructions. The isolated primary neutrophils were then cultured in 12-well plates overnight. Subsequently, they were pre-treated with different doses (5, 10 and 20 μM) of the PARP1 inhibitor (AG14631) for 2 hours before being stimulated with LPS (100 ng/mL). After 24 hours of stimulation, the supernatant was collected for detection of IL-6, IL-10, IFN-γ, TNF-a, and CCL2 expression using cytometric bead array.

### Cytometric Bead Array (CBA)

A Cytometric Bead Array (CBA) Flex Set kit (BD Biosciences, San Jose, CA, United States) was employed to measure levels of interleukin-6 (IL-6), IL-10, interferon gamma (IFN-γ), TNF-a, and C-C motif chemokine ligand 2 (CCL2) in the serum following the manufacturer’s instructions. Samples were analyzed using FACS Canto II Flow Cytometry system (BD Biosciences, USA), and concentrations were determined based on Pharmingen standard curves with FACP Array V3 software (BD Biosciences).

### Flow Cytometry

The prostate tissue was dissected and digested following our established protocol [19]. Briefly, the prostate tissue was placed in 1640 medium supplemented with 2% FBS and 1% penicillin-streptomycin, finely dissected, and homogenized to obtain a single-cell suspension. Subsequently, the cells were filtered through a 70 μm membrane to remove any remaining tissue fragments. The cells were then incubated for 25 minutes at 25 °C in the dark with the antibody master mix: anti-CD45 FITC, anti-CD11b APC-R700, anti-F4/80 BV605, anti-Ly6G BV421, the macrophage were incubated for 15 minutes at room temperature with the antibody master mix: anti-CD45-PE-Cy7, anti-CD11b AF700, anti-F4/80 PE, anti-CD86 BV510, and CD206 AF647 (all antibodies purchased from BD Bioscience, San Jo, CA, USA). After washing twice with FACS buffer, the samples were sorted using FACS Canto II Cytometry (BD Biosciences) and the data were analyzed in FlowJo 10.8.1.

### Hematoxylin-Eosin Staining of Prostate Tissue

The prostate tissues were fixed in 10% formaldehyde for 24 hours, dehydrated, and then embedded in paraffin. Subsequently, they were sliced into 4 μm sections and subjected to hematoxylin and eosin staining following the standard protocol. The sections were imaged using a NanoZoomer Digital Slide Scanner (NDP; Nikon, Tokyo, Japan) and analyzed using NDP view software.

### Quantitative Real-Time PCR

Total RNA was extracted from cells and tissues using the TRIzol reagent (Invitrogen). The RNA concentration was determined using a Thermo Scientific ™ Nanodrop™ system, followed by reverse transcription with a PrimeScript RT reagent kit (Takara) according to the manufacturer’s protocols. Subsequently, cDNA was amplified using SYBR® Green Real-time PCR Master Mix (Takara) on a 7900HT Fast instrument (Applied Biosystems, USA). Primer sequences are listed: TNF-α, forward 5’-CTGAACTTCGGGGTGATCGG-3′, reverse 5′ -GGCTTGTCACTCGAATTTTGAGA −3′; IL-6, forward 5’-CTGCAAGAGACTTCCATCCAG-3′, reverse 5′ -AGTGGTATAGACAGGTCTGTTGG-3′; iNOS, forward 5’-AACGCTTCACTT

CCAATGCAAC-3′,reverse 5′ -CAGCCTCATGGTAAACACGTTC-3′; NF-κB, forward 5’-TGGACGACTCTTGGGAGAAGG-3′,reverse 5′ -AACACAGGCTCATACGGTTTCC-3′; β-actin, forward 5’-GTGACGTTGACATCCGTAAAGA-3′, reverse 5′ -GCCGGACTCATCGTA

CTCC-3.The relative fold gene expression of samples was calculated using the 2^-ΔΔCt^ method.

### Western Blot

Approximately 50 mg of prostate tissue was homogenized and lysed with 350 μL of lysis buffer containing phenylmethyl sulfonylfluoride (PMSF, 1:100). The mixture was then centrifuged at 12,000 rpm for 15 minutes at 4 °C, and the supernatant was collected. Protein concentrations were determined using a bicinchoninic acid (BCA) protein concentration determination kit. Subsequently, the protein samples were combined with 5 × loading buffer, heated in a boiling water bath at 100 °C for 15 minutes. The total protein samples were separated by SDS gel electrophoresis on an 8% or 10% gel based on the molecular weight of the proteins, and transferred to a polyvinylidene fluoride (PVDF) membrane (Millipore, Bedford, USA). Following this step, the membranes were blocked with 5% skim milk in tris buffer solution (TBS) for 2 hours. The primary antibodies (IL-6, HuaBio，Cat: R1412–2,1: 1000；NF-κB P65, Cell Signaling Technology, Cat: 6956, 1: 1000; Phospho-NF-κB P65, ABclonal, Cat: AP1294, 1: 1000; PARP, Cell Signaling Technology, Cat:9542 s, 1: 1000; β-actin, HuaBio, Cat: EM21002, 1: 1000) were added and incubated overnight at 4 °C. Afterward, the membranes underwent five washes with TBS for five minutes each time before being incubated with secondary antibodies at room temperature for two hours; they were then washed again five times for five minutes each time and treated with HRP chemiluminescence solution (Millipore Corporation, Billerica MA., USA). Protein visualization was conducted using an ultrasensitive chemiluminescence imaging system (A Biotechne Brand), followed by quantitative analysis using AlphaView-FluorChem FC3 version3.4.0 software (ProteinSimple, Silicon Valley California). All protein bands were normalized to β-actin content.

### Statistical Analyses

All data are presented as the mean ± SEM. Statistical analyses were performed using SPSS 22.0 software. Statistical significance among groups was evaluated using one-way analysis of variance (ANOVA). *p* value <0.05 was considered statistically significant.

## Results

### Determination of the Dosage Required to Induce Prostatitis in Mice

**Fig. 1:**
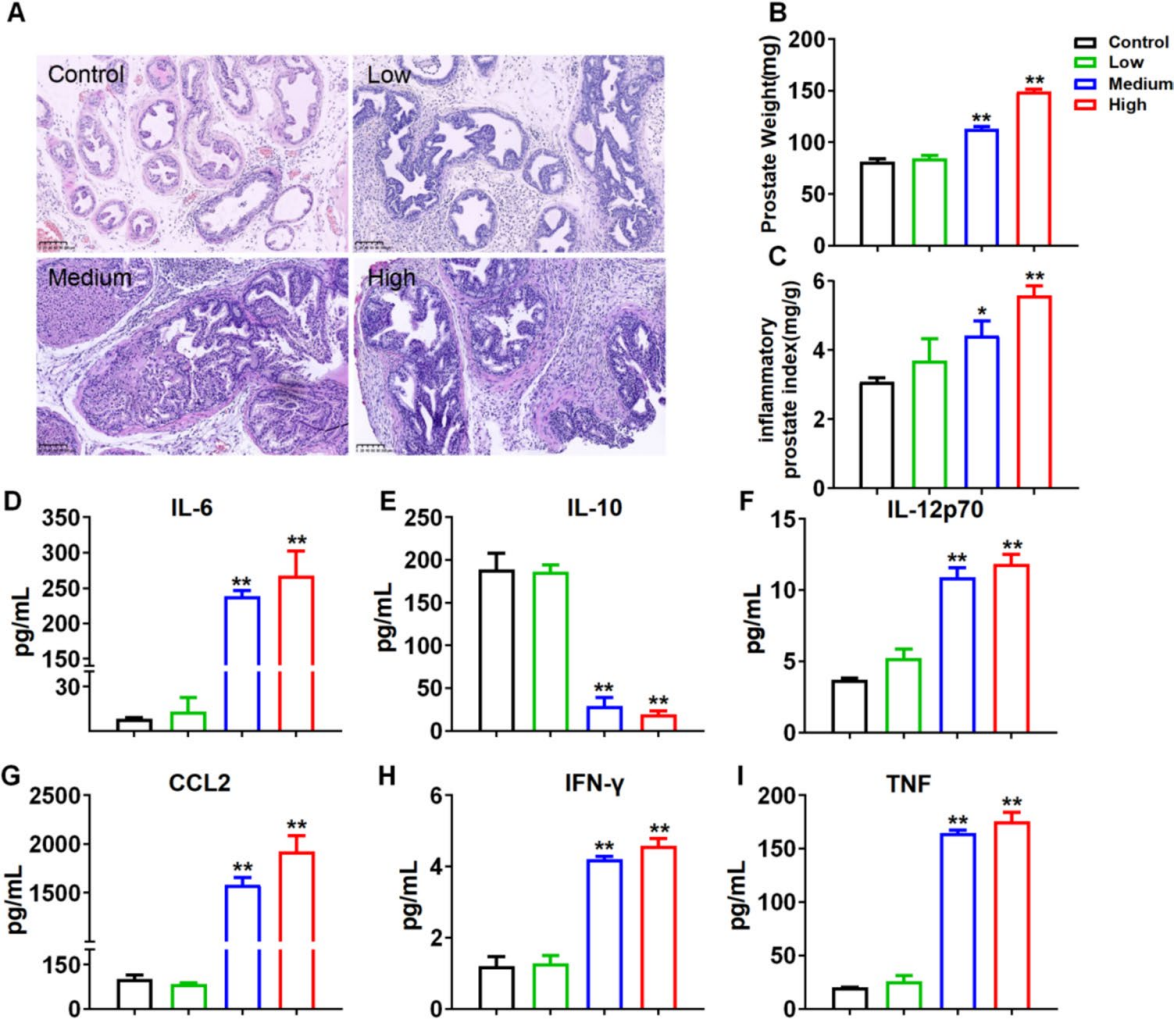
Determination of the dosage required to induce prostatitis in mice. **A** prostate tissue was locally injected with different volumes 1% carrageenan (5, 10, 20 μL) on day 1, and prostate was collected on 7 days, then the representative histological images of HE stained prostate tissues were showed. **B** inflammatory prostate index and (**C**) prostate weight of control, low, middle and high groups. The levels of (**D**) IL-6, (**E**) IL-10, (**F**) IL-12p70, (**G**) IFN-γ, (**H**) CCL2 and (**I**) TNF in the prostate tissue were detected using a CBA kit. The magnification ratio for local pathology was 100×. Data are presented as the means ±SEM (*n* = 6); *p < 0.05, **p < 0.01 *vs.* control group

In this study, we administered varying volume (5, 10, 20 μL) of 1% carrageenan to establish a prostatitis model, to investigated the most effective modeling dose. 1% carrageenan was injected on the first day, and prostate tissue was harvested on day 7 for the assessment of inflammatory damage. Initially, we performed HE staining on prostate tissue to detect pathological injury. Compare to control group, in the Low group, the prostatic epithelium exhibited a cubic to short columnar morphology, arranged in a single layer with neat organization, devoid of papillae. The basal membrane remained relatively intact, and the glandular lumen appeared regular and flat with only slight dilation. There was no evident fibrous connective tissue proliferation or inflammatory cell infiltration within the stroma, nor any significant vascular congestion or necrosis. In the meddle groups, distinct modifications were observed, encompassing columnar or hypercolumnar epithelium with disordered arrangement forming a stratified or pseudostratified layer. The gland presented papillary protrusions into the lumen, accompanied by active proliferation of epithelial cells. Furthermore, inflammatory cell infiltration and fibrous connective tissue proliferation were conspicuous in the interstitium, along with notable vascular congestion. No significant pathological differences were observed between the high-dose and medium-dose groups (Fig. 1A). Moreover, we observed that prostate weight and inflammatory prostate Index were significantly higher in both medium and high groups compared to the control group (Fig. 1B-C). Additionally, the concentrations of IL-10, IL-6, MCP-1, TNF, IFN-γ, and IL-12p70 within prostate tissue were evaluated by the CBA assay. The outcomes demonstrated that no discrepancies in IL-10 level were identified between the low or middle groups in contrast to the control; nonetheless, both the middle and high groups presented significantly lower concentrations than the control. On the contrary, the concentrations of IL-6, MCP-1, TNF-a, IFN-γ, and IL-12p70 were significantly elevated in the middle and high groups compared to the control. Moreover, no differentiations were observed between the middle and high groups (Fig. 1D-I). Based on these discoveries, it is concluded that an administration of 10 μL of a 1% carrageenan saline solution constitutes an optimal dosage for inducing prostatitis.

### PARP1 Exacerbated Prostate Damage in Prostatitis Triggered by Carrageenan

**Fig. 2:**
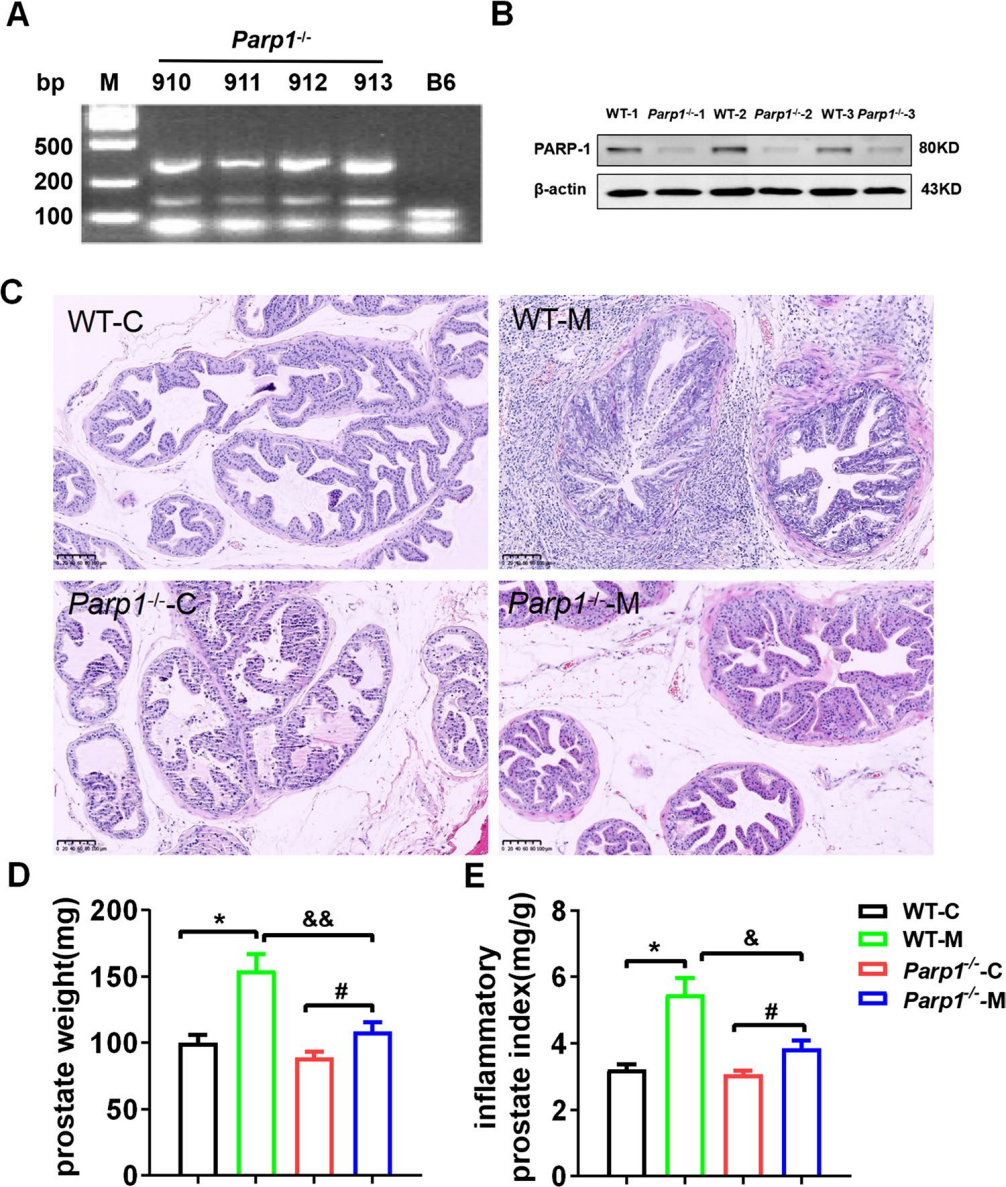
PARP1 exacerbated prostate damage in prostatitis triggered by carrageenan. **A** genotype identification of mice, expected results: Mutant = 350 bp; heterozygote = 112 bp and 350 bp; wild type = 112 bp, notes: A non-specific artifact band appears at ~160 bp. **B** PARP1 protein expression in prostate tissue in *PARP-1*^+/+^ (*WT*) and *PARP-1*^−/−^ mice, detected using western blot. **C** prostate tissue was locally injected with 1% carrageenan (10 μL) on day 1 in *Parp-1*^−/−^ and *WT* mice, and prostate was collected on 7 days, then the representative histological images of HE stained prostate tissues were showed. **D** prostate weight and **E** inflammatory prostate index of *Parp-1*^−/−^ and *WT* mice. The magnification ratio of local pathology was 100×. Data are presented as the means ±SEM (*n* = 6); *p < 0.05 *vs.* WT-C group, ^#^*p* < 0.05 *vs. Parp1*^−/−^ C group, ^&^*p* < 0.05, ^&&^*p* < 0.01 *vs.* WT-M group

Firstly, PARP-1 knockout mice were identified through genotyping (Fig. 2A). Subsequently, the expression levels of PARP-1 in the prostate tissues of both PARP-1 knockout (PARP-1^−/−^) and wild-type (PARP-1^+/+^) mice were assessed using western blot analysis (Fig. 2B). To further explore the function of PARP-1 in prostatitis, *Parp-1*^−/−^ mice were induced with 10 μL of a 1% carrageenan saline solution and inspected for histological alterations and inflammatory index of the prostate. H&E staining revealed that the epithelium of the prostate gland manifested a columnar or hypercolumnar appearance with disordered arrangement, forming a stratified or pseudo-stratified layer group, the gland was papillary and protruded into the gland lumen in *WT* model mice compared with *WT* control mice. Additionally, inflammatory cell infiltration was significantly enhanced in *WT* model mice. Notably, the prostate lesions in the *Parp-1*^−/−^ model group were significantly milder than those in the *WT* model group, suggesting that PARP1 might play a role in regulating the process of prostate inflammation (Fig. 2C).

Furthermore, the prostate weight of the *Parp1*^−/−^ model group was significantly higher than that of the *Parp1*^−/−^ control group but considerably lower than that of the mice in the *WT* model group (Fig. 2D). The results of inflammatory prostate index were in accordance with those of prostate weight (Fig. 2E). Consequently, the findings indicated that PARP1 was capable of positively regulating the progression of prostatitis.

### PARP1 Facilitated the Recruitment of Macrophages and Neutrophils in Prostatitis

**Fig. 3:**
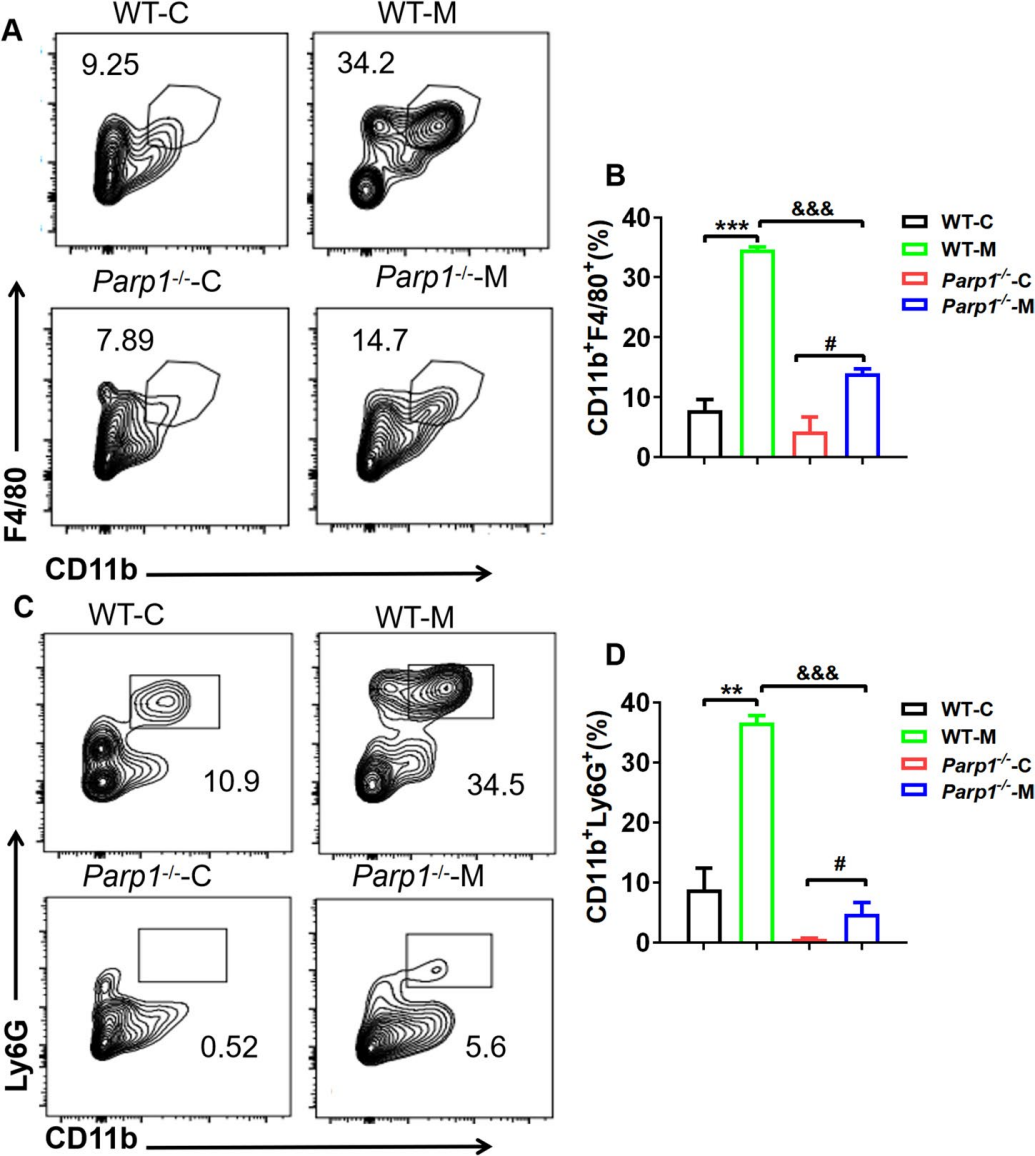
PARP1 facilitated the recruitment of macrophages and neutrophils in prostatitis. Flow cytometric plots (**A**) and quantification (**B**) of macrophage populations (CD45^+^CD11b^+^F4/80^+^) through flow cytometry. Flow cytometric plots (**C**) and quantification (**D**) of neutrophil populations (CD45^+^CD11b^+^Ly6G^+^) through flow cytometry. Data are presented as the means ±SEM (*n* = 6).; ^**^p < 0.01, ^***^p < 0.001 *vs.* WT-C group, ^#^*p* < 0.05 *vs. Parp1*^−/−^ C group, ^&&&^*p* < 0.001 *vs.* WT-M group

Given that neutrophils and macrophages were recruited to the inflammatory site is a crucial factor for the exacerbation of inflammation. Thus, we examined macrophages and neutrophils infiltration within prostate tissue using flow cytometry. The results of macrophages (CD45^+^CD11b^+^F4/80^+^) density in the prostate tissue revealed that prostatitis induced by 1% carrageenan increased the percentage of macrophages in *WT* model group, and also increased in *Parp1*^−/−^ model mice when compared with corresponding control mice. However, the ratio of macrophages significantly in the *Parp1*^−/−^ model group was significantly lower than that in the *WT* model group, suggesting that PARP1 knockout suppressed the recruitment of macrophages in prostatitis (Fig. 3A-B). As shown in Fig. 3C and D, it was observed that the neutrophils (CD45^+^CD11b^+^Ly6G^+^) populations was up-regulated in *Parp1*^−/−^ and *WT* model groups compared to the corresponding control groups, respectively, with a particularly significant decrease observed in the proportion of neutrophils within prostate tissue from *Parp1*^−/−^ model mice. Taken together, these findings demonstrate that PARP1 regulates neutrophils and macrophages recruitment in prostatitis.

### PARP1 Clearly Controlled Inflammation Factors Levels in Prostatitis

**Fig. 4:**
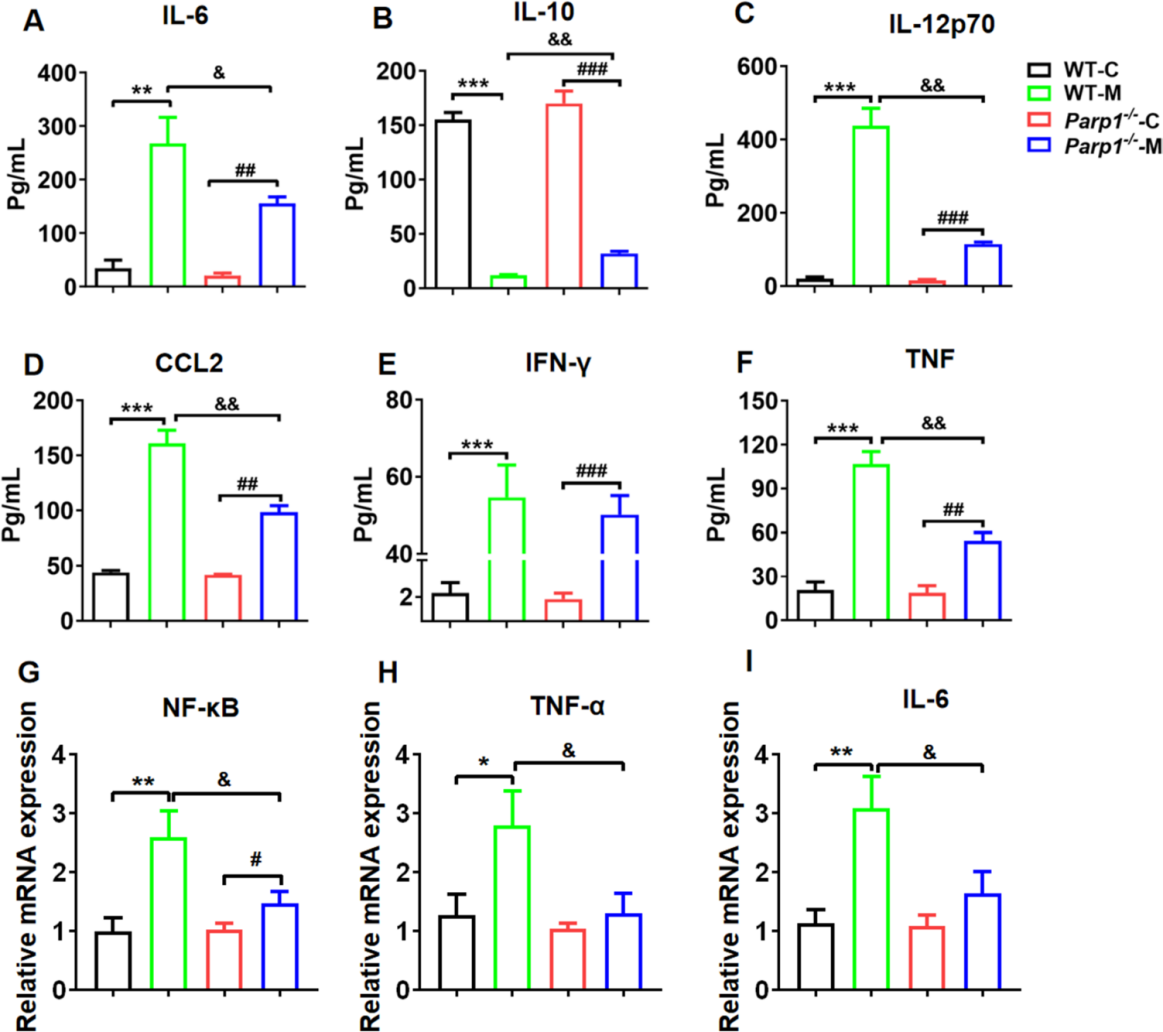
PARP1 clearly controlled the inflammation factors level in prostatitis. **A** IL-6, **B** IL-10, **C** IL-12p70, **D** CCL2, **E** IFN-γ and **F** TNF expression in the prostate tissue of *WT* and *Parp1*^−/−^ mice with or without induction by 1% carrageenan saline solution. **G**-**I** mRNA levels of (**G**) NF-κB, **H** TNF, and **I** IL-6 in the prostate tissue were measured using RT-PCR. Data are presented as the means ±SEM (*n* = 6).; ^**^p < 0.01, ^***^p < 0.001 *vs.* WT control group, ^#^*p* < 0.05 *vs. Parp1*^−/−^ control group, ^&&^*p* < 0.01 *vs.* WT model group

Next, we investigated the expression of inflammatory factors in prostate tissue of *WT* and *Parp1*^−/−^ mice with or without induction by 1% carrageenan saline solution. Results indicated a significant elevation in the levels of IL-6, IL-12p70, CCL2, IFN-γ, and TNF in the *WT* model groups compared to the *WT* control group, while IL-10 declined significantly. Furthermore, IL-6, IL-12p70, CCL2, IFN-γ, and TNF expression also increased in *Parp1*^−/−^ model mice when compared with corresponding control mice, and IL-10 decreased significantly. Additionally, the expression of IL-6, IL-12p70, CCL2 and TNF in the *Parp1*^−/−^ model group was markedly reduced and IL-10 increased significantly compared to that in the WT model group, suggesting that PARP1 enhanced the inflammatory factors secretion in prostatitis. As a pivotal transcription factor, NF-κB facilitates the expression of IL-6 and TNF, which subsequently enhance the activation of NF-κB. Therefore, we detected the mRNA levels of NF-κB, TNF, and IL-6 in prostate tissues *via* fluorescence quantitative PCR. The results indicated that PARP1 enhanced the mRNA levels of TNF, NF-κB, and IL-6 in prostate tissues (Fig. 4G-I). Collectively, these findings indicated that PARP1 plays a crucial role in regulating inflammatory mediators level at the site of inflammation in a validated model of prostatitis.

### PARP1 Modulated the Secretion of Inflammation Factors in Macrophages and Neutrophils Induced by LPS

**Fig. 5:**
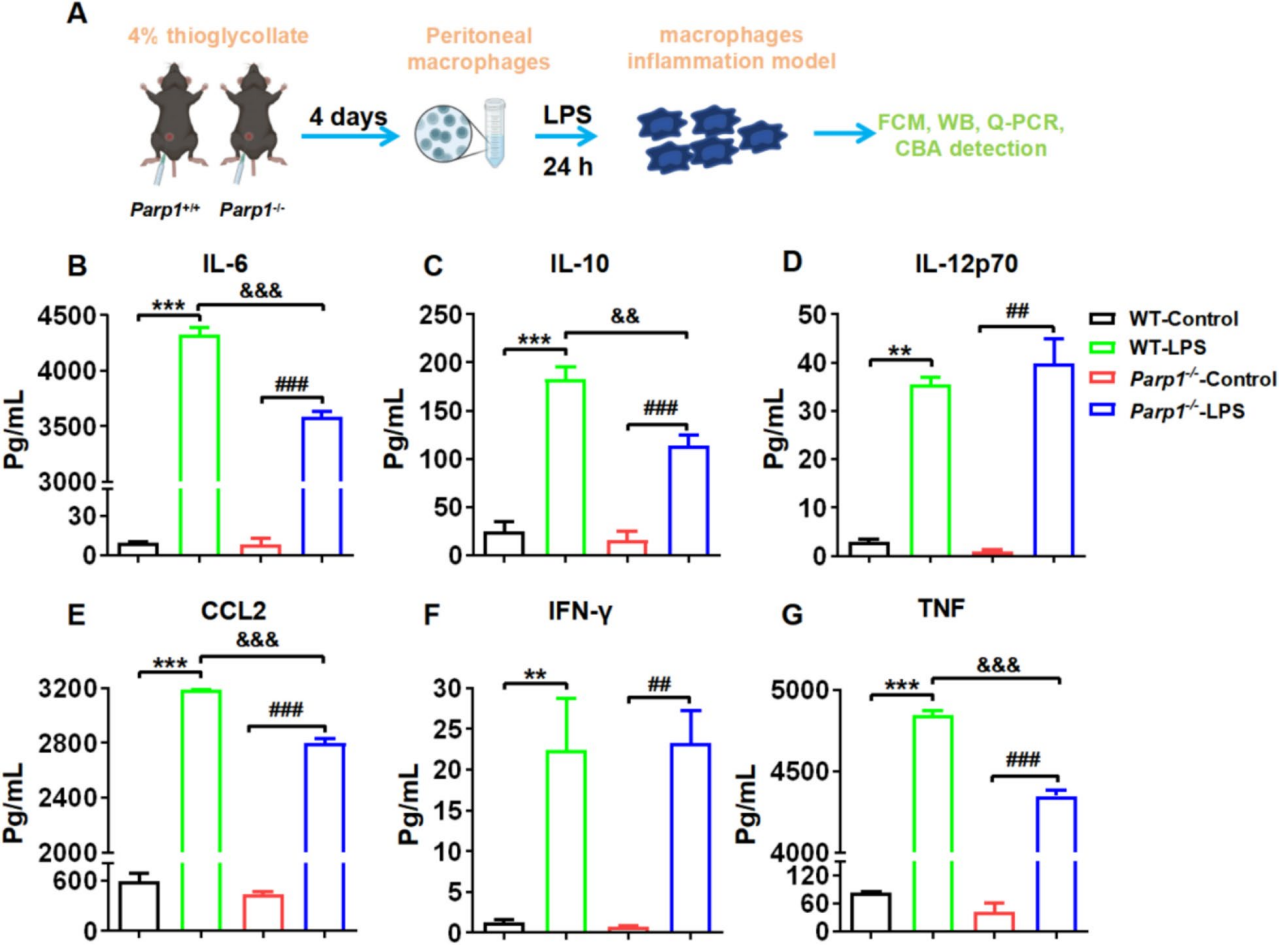
PARP1 modulated the secretion of inflammation factors in macrophages induced by LPS. **A** schematic diagram showing the isolation and treatments of macrophages. Primary peritoneal macrophages were extracted from *WT* and *parp1*^−/−^ mice, then the supernatant was collected after LPS (200 ng/mL) induction for 24 hours, the expression (**B**) IL-6, (**C**) IL-10, (**D**) IL-12p70, (**E**) CCL2, (**F**) IFN-γ and (**G**) TNF were determined by CBA assay. Data are presented as the means ±SEM (*n* = 6); ^**^p < 0.01, ^***^p < 0.001 *vs.* WT control group, ^#^*p* < 0.05 *vs. Parp1*^−/−^ control group, ^&&^*p* < 0.01 *vs.* WT model group

Previously, it was discovered that both macrophages and neutrophils exhibited a significant reduction in the *Parp1*^−/−^ mice prostatitis model induced by 1% carrageenan through flow cytometry (Fig. 3). Subsequently, we further explored which of macrophages or neutrophils played a more crucial role in the PARP1-regulated prostatitis model. Firstly, primary macrophages and neutrophils were extracted *in vitro*, and the effects of PARP1 on the secretion of inflammatory factors in macrophages and neutrophils were investigated by the LPS-induced inflammation model *in vitro*. Primary peritoneal macrophages were extracted from *WT* and *parp1*^−/−^ mice, then the supernatant was collected to determine the levels of inflammatory factors after LPS (200 ng/mL) induction for 24 hours (Fig. 5A). The results indicated that in the LPS-induced macrophage inflammation model, the levels of inflammatory cytokines such as IL-6, IL-10, IL-12p70, CCL2, IFN-γ, and TNF were significantly elevated in macrophages from both *WT* and *parp1*^−/−^ mice. Nevertheless, in comparison with the macrophage from *WT* mice induced by LPS, IL-6, IL-10, CCL2, and TNF were conspicuously decreased in the macrophage from *parp1*^−/−^ mice induced by LPS (Fig. 5B-G).

**Fig. 6:**
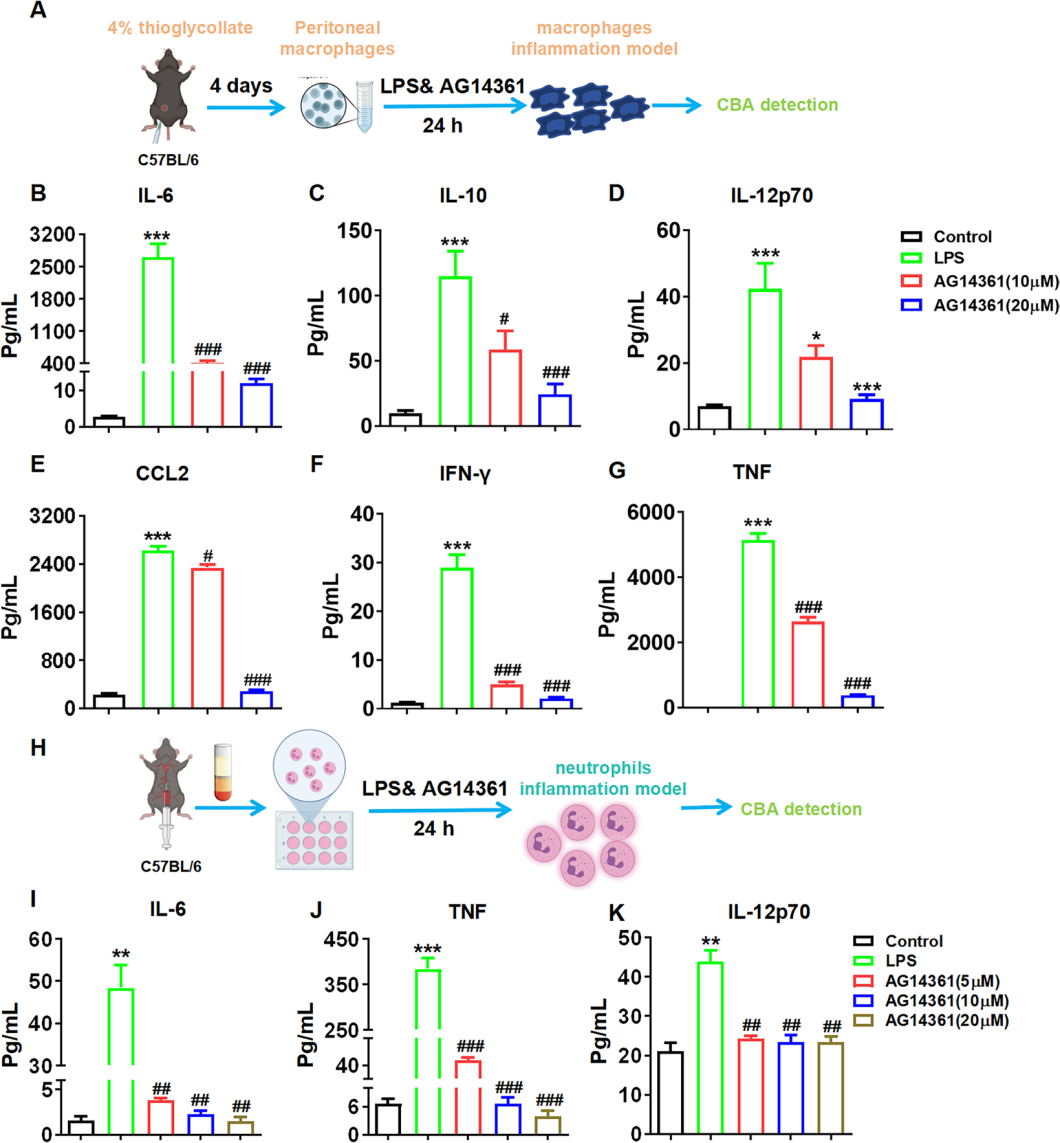
PARP1 modulated the secretion of inflammation factors in neutrophils induced by LPS. **A** schematic diagram showing the isolation and treatments (PARP1 inhibitors (AG14361)) of macrophages from *WT* mice, then the supernatant was collected after LPS (100 ng/mL) induction for 24 hours. The expression (**B**) IL-6, (**C**) IL-10, (**D**) IL-12p70, (**E**) CCL2, (**F**) IFN-γ and (**G**) TNF were determined by CBA assay. **H** primary neutrophils were isolated from blood and exposed to PARP1 inhibitors (AG14361) for 24 hours in a model of LPS-induced (100 ng/mL) inflammation. Subsequently, the expression (**I**) IL-6, (**J**) TNF and (**K**) IL-12p70 in the supernatant were quantified using a CBA assay. Data are presented as the means ±SEM (*n* = 6); ^**^p < 0.01, ^***^p < 0.001 *vs.* WT control group, ^#^*p* < 0.05 *vs. Parp1*^−/−^ control group, ^&&^*p* < 0.01 *vs.* WT model group

As illustrated in Fig. 6B-G, the results indicated that the levels of inflammatory cytokines, including IL-6, IL-10, IL-12p70, CCL2, IFN-γ, and TNF, were significantly reduced in a dose-dependent manner in macrophages following treatment with PARP1 inhibitors (AG14361) in an LPS-induced model. Additionally, we extracted primary neutrophils from blood and treated the cells with PARP1 inhibitors (AG14361) for 24 hours in a model of LPS-induced inflammation (Fig. 6H). Results showed that the PARP1 inhibitor prominently reduced the secretion of IL-6, TNF, and IL-12p70 (Fig. 6I-K) in a dose-dependent manner. However, the results demonstrated that upon stimulation with LPS to induce inflammation in both macrophages and neutrophils, the secretion level of inflammatory factors in macrophages was significantly higher than that of neutrophils (*e.g.*, IL-6 by 50 times, TNF by 12 times). As consequence, macrophages exerted a more crucial role in the regulation of inflammatory models.

### PARP1 Promoted the Inflammatory Response *Via* the TNF/NF-κB Pathway in Macrophages Stimulated by LPS

**Fig. 7:**
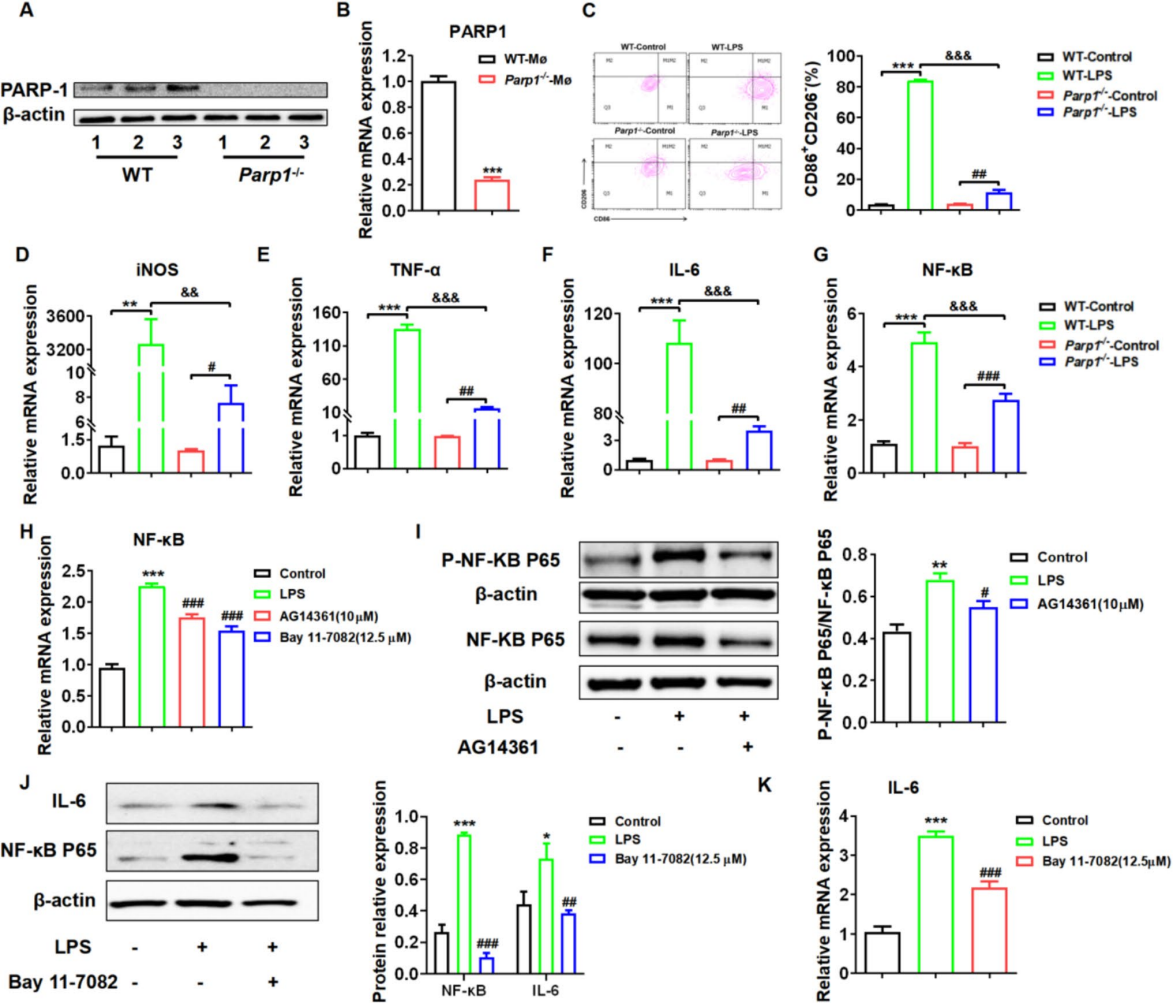
PARP1 promoted the inflammatory response *via* the TNF/NF-κB pathway in macrophages stimulated by LPS. **A** PARP1 protein (**B**) and mRNA levels were detected in peritoneal murine macrophages from *WT* (*Parp1*^+/+^) and *Parp1*^*−/−*^ mice using western blot and RT-qPCR experiment, respectively. **C** flow cytometric plots and quantification of CD86^+^ macrophage ratio in macrophages using flow cytometry. RT-qPCR were employed to detect the mRNA levels of (**D**) iNOS, (**E**) TNF-α, (**F**) IL-6, and (**G**) NF-κB in macrophages from *WT* and *Parp1*^*−/−*^ mice stimulated by LPS. (H)NF-κB mRNA levels were quantified in macrophages treated with PARP1 inhibitor (AG14361) or NF-κB inhibitor (bay 11–7082) using RT-qPCR experiment. (I) the representative images and quantitative statistics of P-NF-κB P65, NF-κB P65 protein expression in macrophages treated with PARP1 inhibitor was determined using western blot. (J) the representative images and quantitative statistics of NF-κB and IL-6 protein expression in macrophages treated with NF-κB inhibitor was determined using western blot. (K) IL-6 mRNA levels were quantified in macrophages treated with NF-κB inhibitor using RT-qPCR experiment. Data are presented as the means ±SEM (*n* = 6); ^**^p < 0.01, ^***^p < 0.001 *vs. WT* control group, ^#^*p* < 0.05 *vs. Parp1*^−/−^ control group or *WT* model group, ^&&^*p* < 0.01 *vs. WT* model group

Macrophages exhibit a significant degree of functional diversity and play a vital role in the normal development, homeostasis, tissue repair, and immune response against pathogens within the body. We independently isolated peritoneal murine macrophages from *WT* (*Parp1*^+/+^) and *Parp1*^*−/−*^ mice. Our findings revealed that the PARP1 protein and mRNA levels were significantly reduced in macrophages derived from *Parp1*^*−/−*^ mice compared to those from *WT* mice (Fig. 7A-B). Additionally, we established an M1 polarization model of macrophages induced by LPS to investigate the regulatory role of PARP1 in macrophage inflammation. According to the literature, the CD206^−^CD86^+^ macrophage population is regarded as an M1-like phenotype. As depicted in Fig. 7C, CD206^−^CD86^+^ M1 macrophages are significantly down-regulated in the *Parp1*^−/−^ macrophage inflammation model. Additionally, results demonstrated that the level of iNOS, IL-6 and TNF-α mRNA levels were also significantly down-regulated in the *Parp1*^−/−^ macrophage inflammation model (Fig. 7D-F), which are M1 phenotype markers. These results suggested that PARP1 promoted the expression of the CD86 co-stimulatory molecule on the surface of M1 macrophages and the intermediate product iNOS, along with cytokines such as IL-6 and TNF-α.

**Fig. 8:**
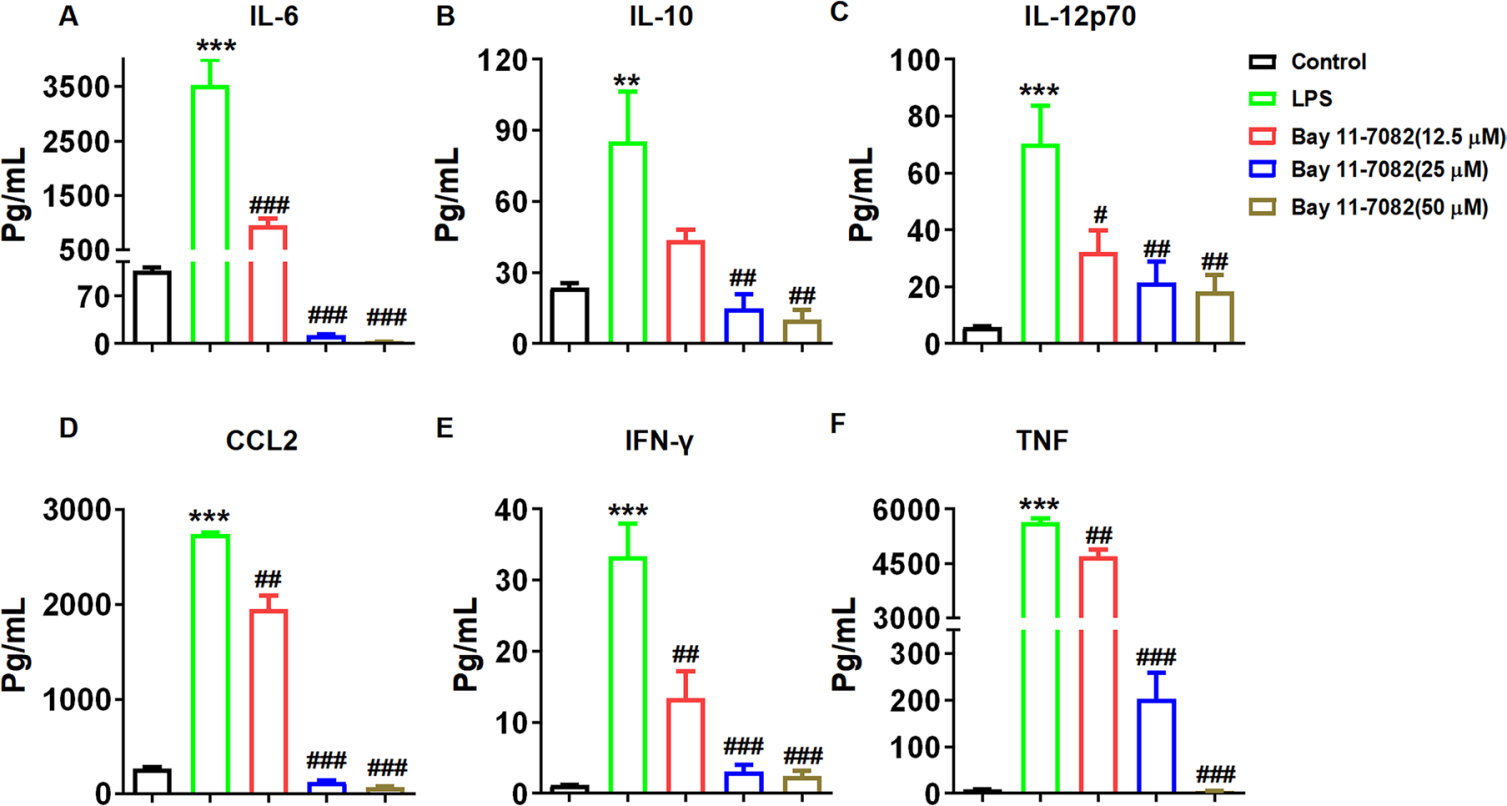
NF-κB facilitated the inflammatory secretion in macrophages stimulated by LPS. The supernatant was collected following the intervention of the NF-κB inhibitor (bay 11–7082) during a 24-hour induction of macrophages from *WT* mice with LPS (100 ng/mL). The expression level (**A**) IL-6, (**B**) IL-10, (**C**) IL-12p70, (**D**) CCL2, (**E**) IFN-γ and (**F**) TNF in the supernatant were quantified using a CBA assay. Data are presented as the means ±SEM; ^***^p < 0.001 *vs.* control group, ^#^*p* < 0.05，^###^*p* < 0.001 *vs.* model group (*n* = 6)

TNF-α is a crucial cytokine that triggers NF-κB activation *in vivo*. Excessive and persistent NF-κB activation functions as a vital mediator in facilitating inflammatory diseases. The results demonstrated that the level of NF-κB mRNA in the model group was significantly higher compared to that in the control group, and the expression of NF-κB mRNA increase in *Parp1*^−/−^ macrophages induced by LPS was significantly reduced (Fig. 7G). Simultaneously, it was observed that the intervention with PARP1 inhibitors led to a significant downregulation of NF-κB mRNA levels (Fig. 7H). Additionally, both P-NF-κB P65 and NF-κB P65 protein levels were reduced. The decreased P-NF-κB P65/NF-κB P65 ratio suggests that PARP1 plays a regulatory role in NF-κB activation (Fig. 7I). AS a result, the IL-6 protein and mRNA expression was obviously decreased after NF-κB inhibitors (Bay 11–7082) intervention (Fig. 7J-K). As showed in Fig. 8, the results demonstrated that the levels of inflammatory cytokines, including IL-6, IL-10, IL-12p70, CCL2, IFN-γ, and TNF, were markedly reduced in a dose-dependent manner in macrophages treated with NF-κB inhibitors in an LPS-induced model.

The collective effect of these molecules attracts other immune cells and chemokines to the inflammation site, thereby enhancing the inflammatory response. Consequently, PARP1 might enhance LPS-induced inflammatory responses in macrophages through the NF-κB pathway.

## Discussions

PARP1 (poly(ADP-ribose) polymerase 1 has been extensively investigated for its regulatory role in a variety of inflammatory diseases, including rheumatoid arthritis, systemic lupus erythematosus, inflammatory bowel disease, and acute lung injury [20]. This study probed into the regulatory role of PARP-1 in prostatitis. Our results reveal that PARP1 aggravated prostatitis, and promoted macrophage and neutrophil infiltration at inflammatory sites and upregulates the expression of inflammatory cytokines such as IL-6, CCL2, and TNF. The main potential mechanism by which PARP1 might regulate the secretion of inflammatory factors in M1-like macrophages through NF-κB signaling to alleviate carrageenan-induced prostatitis.

Neutrophils and macrophages are pivotal in the modulation of prostatitis, influencing its development and progression through the secretion of extracellular vesicles, regulation of inflammatory factor expression, and interaction with other immune cells [21, 22]. The prolonged generation of multiple proinflammatory cytokines triggers the activation of neutrophils and macrophages, initiating a cascade of proinflammatory factor secretion that culminates in an ‘inflammatory storm’ [23]. In the present study, we witnessed a remarkable recruitment-enhancing effect of PARP1 on both macrophages and neutrophils in the prostatitis model, and significantly higher expression levels of pro-inflammatory cytokines IL-6, IL-12p70p70, CCL2, and TNF, while the expression of the anti-inflammatory cytokine IL-10 was notably lower. Thus, the recruitment of neutrophils and macrophages to the inflammatory site is a vital factor for exacerbating inflammation; the sustained increase of inflammatory factors can deteriorate symptoms.

Subsequently, we isolated primary macrophages and neutrophils *in vitro* to compare the secretion of inflammatory mediators within the LPS-induced inflammatory model, revealing that macrophages exert a more pronounced regulatory effect on inflammation model. PARP1 significantly augmented levels of pro-inflammatory factors such as IL-6, IL-10, CCL2 and TNF. Considering that CCL2 is a pivotal chemokine involved in the recruitment of macrophages to inflammatory sites [24], it was also observed that CCL2 levels were significantly reduced in the prostate tissue of *Parp1*^−/−^ mice. PARP1 might promotes CCL2 secretion in prostate tissue to recruit macrophages, subsequently intensifying inflammatory damage through increased macrophage secretion of pro-inflammatory factors.

Macrophages are plastic, and can be polarized into M1-like or M2-like phenotypes by pro-inflammatory stimuli (*e.g.*,IFN-γ and LPS) or anti-inflammatory stimuli (*e.g.*, IL-4 and IL-13). M1 macrophages are are mainly associated with the pro-inflammatory response. Activated M1 macrophages can exacerbate tissue damage and secrete a diverse array of inflammatory mediators [25, 26]. A variety of inflammatory diseases can be ameliorated by inhibiting M1-type macrophage polarization. It is documented that inhibition of macrophage M1 polarization in DSS mice improved colitis [27]. And periodontitis disease progression was inhibited by reducing M1 macrophage polarization and decreasing the iNOS, TNF-α, and IL-6 expression [28]. *In vitro* studies show that PARP1 significantly enhances macrophage polarize toward CD86^+^CD206^−^ M1 macrophage. The inducible nitric oxide synthase (iNOS), which is highly expressed after polarization into M1 macrophages, promotes the release of various inflammatory cytokines and chemokines, attracting other immune cells to inflammation sites [29]. Our findings indicated that PARP1 markedly elevated the expression levels of iNOS, IL-6, and TNF mRNA, as well as significantly enhanced the secretion of inflammatory cytokines including IL-6, IL-10, IL-12p70, CCL2, IFN-γ, and TNF.

In recent years, numerous studies have shown that NF-κB is a probal target for malignant (prostatic carcinoma) and benign (prostatic inflammation) prostatic conditions, however, its role in these diseases exhibits considerable diversity. It has reported that CCL5/CCR5/SHP2 axis sustains Stat1 phosphorylation and activates NF-κB signaling promoting M1 macrophage polarization and exacerbating chronic prostatic inflammation [30]. Pseudomonas may promote the progression of benign prostatic hyperplasia through LPS activation of NF-κB signaling [31]. PARP-1 has been reported to be a coactivator of NF-κB and is involved in NF-κB-dependent transcriptional regulation [32]. In this study, we further confirm that PARP-1 activates the NF-κB pathway in macrophages, leading to prostatitis. Conversely, inhibiting or knocking down PARP-1 activity in macrophages alleviates prostatitis. It is important to note, however, that activation of the NF-κB signaling pathway plays a crucial role in tumor immunity. Research has demonstrated that cellular vesicles secreted by prostate cells, which contain TRPM8 RNA, can activate TLR3/NF-κB-mediated inflammatory signaling upon endocytosis by prostate epithelial cancer cells. This process degrades the extracellular matrix, promotes NK cell infiltration, and inhibits prostate cancer progression [33]. Collectively, NF-κB exhibits dual effects in different disease models or inflammatory settings.

**Fig. 9:**
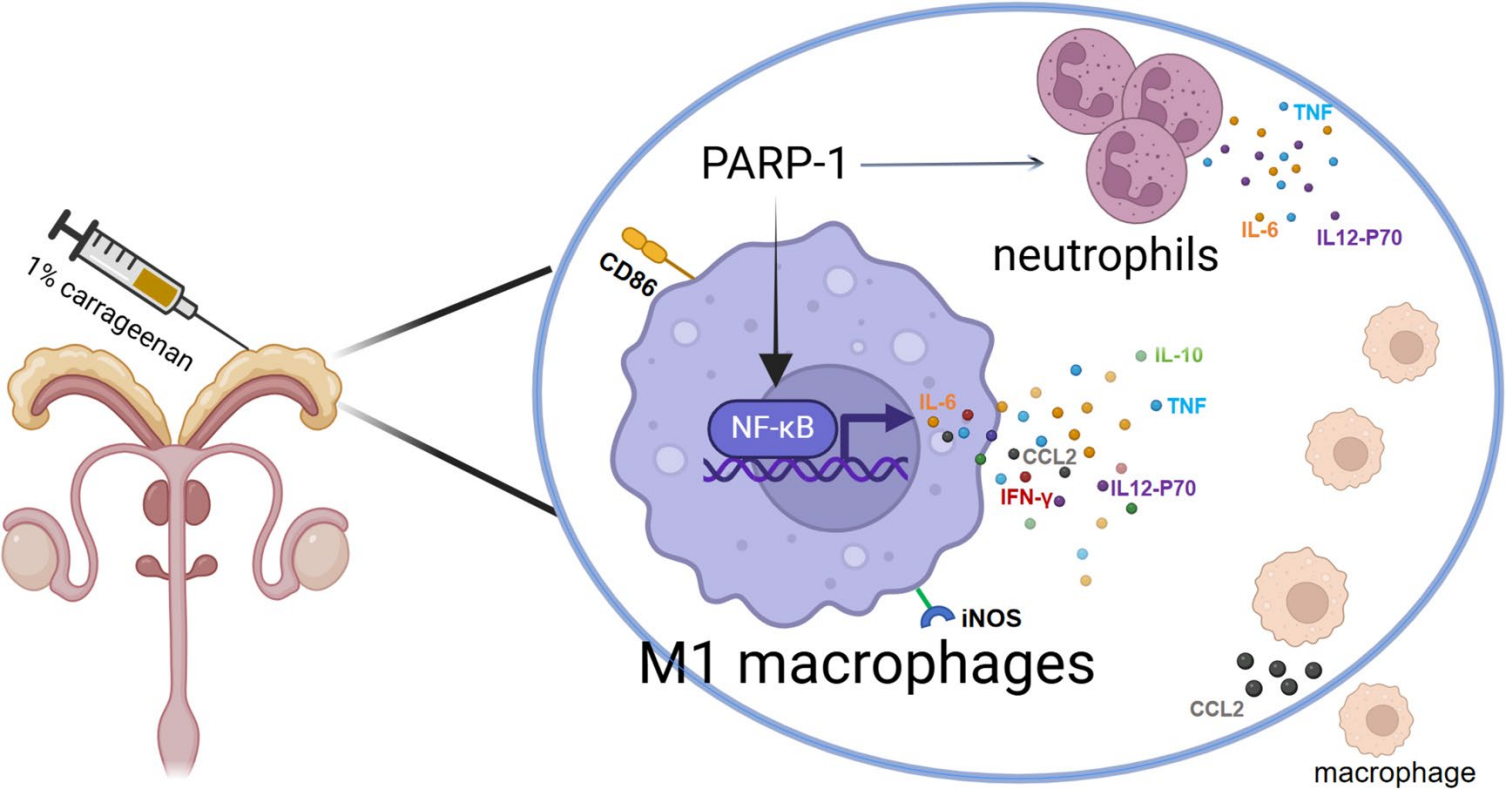
A schematic illustration of the mechanism through which PARP1 promote prostatitis by driving macrophage polarization toward M1 macrophages

In conclusion, we discovered that PARP1 could exacerbate prostatitis by facilitating macrophage infiltration and differentiation to the M1 phenotype. Mechanistically, PARP1 promote the NF-κB expression, which could regulate downstream inflammatory cytokines, including IL-6, IL-10, IL-12p70, CCL2, IFN-γ in M1 macrophages, leading to the accumulation of proinflammation factors and ultimately exacerbating prostatitis. A schematic illustration of the results of this study is shown in Fig. 9. Collectively, our study provides a theoretical basis for new applications of PARP1 inhibitor, which could be a possible new option for the treatment of prostatitis.

## Supplementary Information

ESM 1 (DOCX 4513 kb)

## Data Availability

No datasets were generated or analyzed during the current study.
