# Supplementary material for: PARP1 Exacerbates Prostatitis by Promoting M1 Macrophages Polarization through NF-κB Pathway
Source: Inflammation. 2025 Mar 4;48(5):3022–35. doi: 10.1007/s10753-025-02247-y (PMC12596315; doi:10.1007/s10753-025-02247-y)
Supplement: Supplementary file 1 — (DOCX 4513 kb) [file 10753_2025_2247_MOESM1_ESM.docx]

**S1**

**
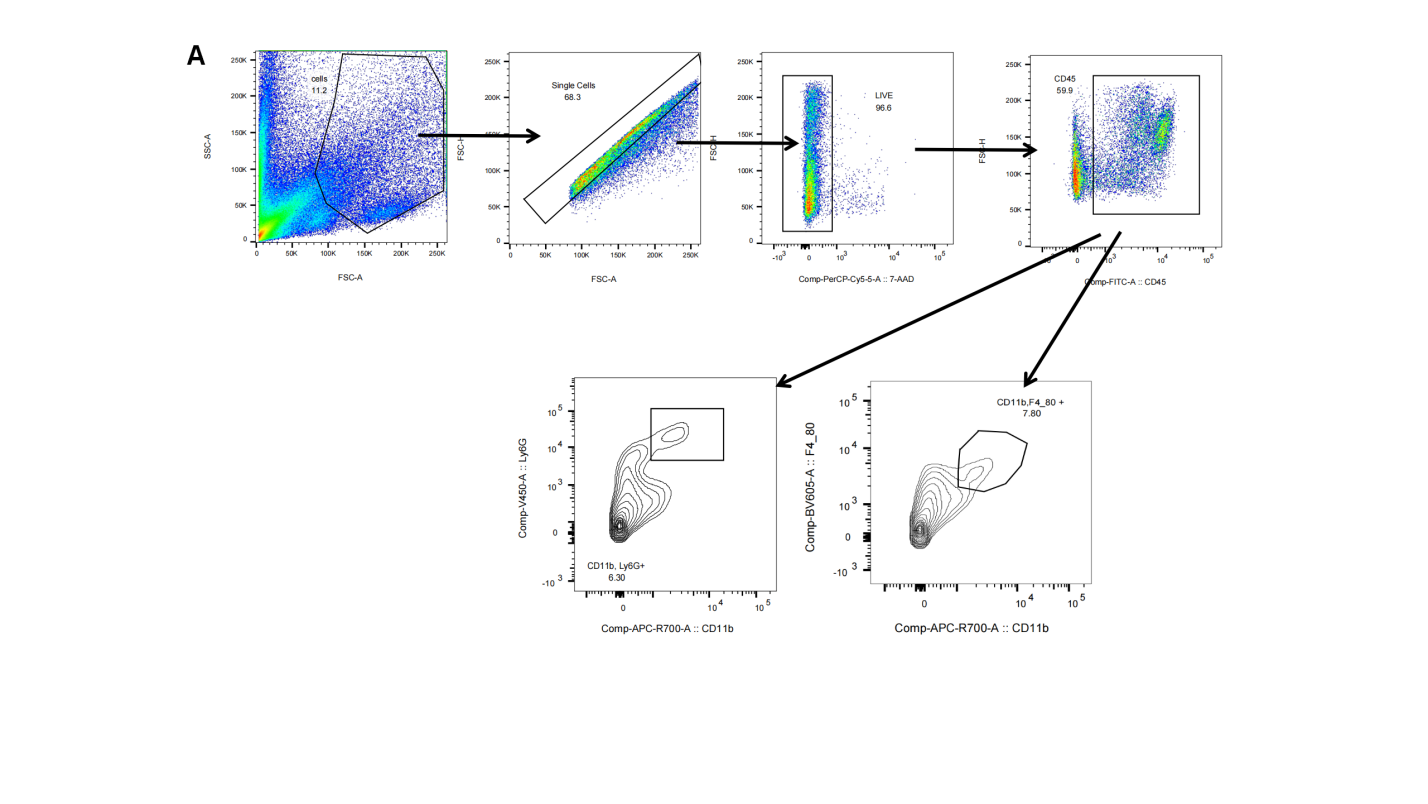
**

Figure S1. Flow cytometry characterization of immune populations in prostatic tissue, related to Figures 3. (A)The gating strategy for myeloid flow cytometry staining panels is illustrated. Arrows denote the parent population that the subsequent plot is gated on. Gates were drawn manually based on fluorescence minus one (FMO) controls.
